# Reproductive health concerns of women living with sickle cell disease

**DOI:** 10.1002/hem3.70167

**Published:** 2025-07-16

**Authors:** Edeghonghon Olayemi

**Affiliations:** ^1^ Department of Haematology University of Ghana Medical School Accra Ghana

With advancements in care, people living with sickle cell disease (SCD) now live to reproductive age and beyond; however, improvements in care for their reproductive health have lagged far behind, leading to excess mortality and morbidity among pregnant women living with SCD compared to those without the disease.^1^ Globally, there is insufficient knowledge regarding reproductive health among women living with SCD. The association between chronic diseases such as SCD and mental health is well known. Unfortunately, women with SCD face additional mental stress, worrying about the risk of passing the disease and/or the sickle cell gene to their children. Three major themes emerged from a new study led by Lisa Roberts,^2^ using a qualitative research approach to investigate reproductive health concerns of women with SCD and sickle cell trait (SCD/T).

## THEME 1: DILEMMA OF LOVE VERSUS RISK

Under this theme the authors outlined the effect of SCD/T on dating, fertility, and family choices. They demonstrated that women living with SCD in the reproductive age bracket are faced with uncertainty about their reproductive future. Choices like romantic relationships and marriage, which their peers without the disease take for granted, can have significant health implications because they have to juggle their desire for fulfilling romantic relationships and procreation with the risks posed by the disease to themselves and their offspring.

## THEME 2: SCD/T KNOWLEDGE

This theme revealed that some participants had insufficient knowledge of SCD/T and the possible effects of SCD/T on reproductive health. As a result, they felt unprepared and had difficulty asking pertinent questions even when they had the opportunity to do so during encounters with health care personnel. On the other hand, those who were knowledgeable about the disease faced the dilemma of when and how to discuss this with a new partner, who may not be as knowledgeable.

## THEME 3: MENTAL AND EMOTIONAL TOLL OF SCD/T

This theme clearly illustrated that while navigating a path from adolescence to young adulthood is stressful, the added burden on young persons living with SCD could lead to mental and emotional challenges. Though provision of adequate knowledge about the disease may help reduce this burden, it may never completely remove the mental health issues leading to depression, isolation, and helplessness. This was encapsulated by a quote from one of the participants who said:

Sickle cell is not just about the pain. It's mentally, it's emotionally. It's the jobs that I was talking to you about. It's about college, school, work. Because it's like you can have a crisis, you can fall behind in school. Then you start to feel less of yourself and feel like you're not smart enough. And the same with jobs. So, I feel like it can really take a toll on your mental and your emotional state if you allow it to.

This mental toll interferes with relationships with partners, peers, and close relatives, the very people needed to provide support during difficult times like sickle cell crises and pregnancy. So, some people living with SCD spend time and energy trying to conceal their symptoms instead of dealing with them. This makes living with SCD even more difficult for both the person living with it and their caregivers.

## CONCLUSION

This study by Roberts and colleagues^2^ lays the foundation for further investigation in this area. It reaffirms the lack of support or the perceived lack of support provided by health care workers to people living with SCD, which is similar to that reported by earlier studies. Although it is not clear why the authors included people with sickle cell trait (HbAS), which is essentially a benign condition, in their study, a possible reason may be the risk of their offspring inheriting the sickle cell gene.^3^ This work exposes the apprehension faced by young women living with SCD/T between their desire to be loved and the potential health complications that may arise from their desire for procreation. This is a complex situation without easy solutions. However, as stated by the authors, it may be helpful for people with SCD to develop an early reproductive life plan. Creating a reproductive life plan allows individuals to make informed decisions about family planning, considering the potential genetic implications of SCD. This proactive approach may help reduce the mental stress associated with chronic diseases like SCD and make them better prepared for future challenges. The cost of some components of a reproductive life plan, such as assisted reproductive technologies, makes them either unavailable or inaccessible to people living with SCD in resource‐challenged countries such as those in sub‐Saharan Africa, where most people with SCD live.^4^ On the other hand, a good place to start may be to focus on areas such as the provision of age‐appropriate reproductive health information and counseling, which are less expensive, more accessible, and acceptable in conservative parts of the world due to cultural and traditional barriers.

## AUTHOR CONTRIBUTIONS

**Edeghonghon Olayemi**: Conceptualization; writing—original draft; writing—review and editing.

## CONFLICT OF INTEREST STATEMENT

The author declares no conflicts of interest.

## FUNDING

This research received no funding.

## Data Availability

Data sharing is not applicable to this article as no new data were created or analyzed in this study.
